# Associations between fucosyltransferase 3 gene polymorphisms and ankylosing spondylitis: A case–control study of an east Chinese population

**DOI:** 10.1371/journal.pone.0237219

**Published:** 2020-08-07

**Authors:** Guangming Jiang, Renfang Han, Mengya Chen, Rui Liu, Meng Wu, Xu Zhang, Yubo Ma, Yaping Yuan, Ran Wang, Zongwen Shuai, Faming Pan

**Affiliations:** 1 Department of Epidemiology and Biostatistics, School of Public Health, Anhui Medical University, Hefei, Anhui, China; 2 Division of Life Sciences and Medicine, Department of Blood Transfusion, The First Affiliated Hospital of USTC, University of Science and Technology of China, Hefei, Anhui, China; 3 Key Laboratory of Anhui Medical Autoimmune Diseases, Hefei, Anhui, China; 4 Department of Rheumatology and Immunology, The First Affiliated Hospital of Anhui Medical University, Hefei, Anhui, China; King Saud University, SAUDI ARABIA

## Abstract

Many susceptibility genes of inflammatory bowel disease (IBD) are associated with ankylosing spondylitis (AS). Fucosyltransferase 2 (*FUT2*) and *FUT3* genes are related to IBD. This study aimed to investigate whether these genes correlated with the susceptibility to AS. Questionnaires of 673 patients with AS, and peripheral blood specimens of the patients and 687 healthy controls were collected. *FUT2* and *FUT3* genes were genotyped using the SNPscan method. Frequency differences of the genes at different levels, haplotypes, and interactions were analyzed. No frequency differences were found between the cases and the controls in all the genotypes and the alleles of rs1047781, rs1800028, rs1800030, and rs812936. For rs28362459, a significant difference in allele frequencies was observed in the total participants between the groups [χ^2^ = 7.515, *P*_corrected_ = 0.030; adjusted odds ratio (OR_adjusted_)_G/T_ = 0.782; 95% confidence interval (CI), 0.650–0.941]. The frequencies of haplotypes TT (rs812936–rs28362459) (*χ*^2^ = 5.663, *P*_permutation_ = 0.039) and TG (rs812936–rs28362459) (*χ*^2^ = 7.456, *P*_permutation_ = 0.013) in the total participants, and TG (rs812936–rs28362459) in the female subgroup (*χ*^2^ = 5.624, *P*_permutation_ = 0.047) showed significant differences between the cases and the controls. No frequency differences at the phenotypic level were found. Two-factor interactions were observed between rs28362459-TG and age, rs28362459-TG and sex, rs28362459 and rs1047781, and the Lewis and secretor status. Rs28362459-G was related to some aggravated symptoms of AS (all *P*_corrected_ < 0.05). These findings indicated that *FUT3* polymorphisms were associated with human predisposition to AS at the allele and haplotype level. Rs28362459-G might decrease the susceptibility to AS, but aggravate relevant symptoms.

## Introduction

Ankylosing spondylitis (AS) is a serum-negative connective tissue disease characterized by back pain and rigidity. Its etiology involves genetic, environmental, infectious, and immune factors. A number of studies have proved that genetic factors play a significant role in the pathogenesis of the disease. At least 36 susceptibility genes of AS have been found so far [1], among which human leukocyte antigen B27 (HLA-B27) has the closest association with the disease [2, 3], especially HLA-B*2705, although HLA-B*2706 and HLA-B*2709 are not related to AS [4–6]. However, these genes can explain only 24.4% of the genetic predisposition toward AS, with HLA-B*27 accounting for 20.1% and the remaining genes for 4.3%. This indicates that more than 75% of AS genetic susceptibility has yet to be found [1, 2, 7].

Some studies suggested that about 50%–60% of patients with AS also suffered from intestinal inflammation [2, 8], and 5%–10% of patients with AS had clinical evidence of inflammatory bowel disease (IBD), including Crohn's disease (CD) and ulcerative colitis (UC) [9, 10]. Another study found that the first-degree relatives of patients with AS were three times more likely to suffer from IBD compared with the general population [1, 11]. A genome-wide association study revealed that 20 of 31 AS-susceptibility genes overlapped with those of IBD; the same single-nucleotide polymorphisms (SNPs) had the same or similar effects in both diseases [1, 7, 12, 13]. In addition, the genes linked to AS involved various aspects of infection and immunology [14]. Therefore, it is promising to further search for susceptibility genes of AS among infection-related genes, especially relevant genes of IBD.

The human fucosyltransferase2 (*FUT2*) gene encodes α1,2-L-fucosyltransferase, which catalyzes the biosynthesis of type-1 H (Le^d^), a blood group antigen, using a type-1 precursor (Le^c^) and fucose. Type-1 H is also a precursor of several other blood group antigens, such as type-1 A or type-1 B. An individual having functional *FUT2* (*Se*) is called a secretor, whose fluids and secretions may contain soluble A, B, and H antigens. Otherwise, an individual with two null *FUT2* alleles (*se*) is called a non-secretor, who cannot synthesize these antigens. The human *FUT3* gene encodes α1,3/4-L-fucosyltransferase, which catalyzes the synthesis of Le^a^ antigen in non-secretors using Le^c^ and fucose, or catalyzes the synthesis of Le^b^ in secretors using type-1 H and fucose. Therefore, non-secretors with functional *FUT3* (*Le*) exhibit the Le(a+b-) serotype, secretors with *Le* exhibit the Le(a-b+) serotype, and individuals with two null *FUT3* alleles (*le*) present the Le(a-b-) serotype despite their secretor status [15]. The type-1 A, B, H, and Lewis antigens (such as Le^b^) are widely distributed in human fluids and secretions and on the surface of genitourinary and gastrointestinal epithelial cells. Hence, they are called histo-blood group antigens (HBGAs).

*FUT2* and *FUT3* genes are closely related to gut inflammation. The expression of both *FUT2* and *FUT3* genes needs the participation of host intestinal flora [16–19]. Meanwhile, non-secretors are more susceptible to CD [16, 20]; *FUT3* gene polymorphisms and expression in the intestinal tract are associated with the susceptibility to UC [21]. Therefore, it is supposed that *FUT2*, *FUT3*, and HBGAs may play certain roles in the pathogenesis of AS and relevant intestinal inflammation. The aim of this study was to explore the associations between *FUT2/FUT3* polymorphisms and AS in an east Chinese population.

## Materials and methods

### Participants

A total of 673 patients with AS were selected at the Clinic of Rheumatology and Immunology, the First Affiliated Hospital of Anhui Medical University from January 2015 to June 2018. All the patients were diagnosed by senior rheumatologists according to the New York Diagnostic Criteria (revised in 1984). Meanwhile, 687 age- and sex-matched healthy controls were recruited from the Health Checkup Center of the hospital. All the participants came from east China, mostly from Anhui Province. They had no underlying diseases such as liver, kidney, and cardiovascular or cerebrovascular diseases. In addition, they had not suffered from infectious diseases, such as acute intestinal inflammation, within six months before sampling; had not undergone surgery within one year; and had not taken non-steroidal anti-inflammatory drugs, glucocorticoids, traditional Chinese medicine, and other anti-rheumatic drugs within three months prior to sampling. Furthermore, none of the healthy controls had family history of rheumatic and autoimmune diseases. All the participants were informed of the study in detail and voluntarily signed a written informed consent prior to their participation. Each of the patients with AS then filled in a questionnaire on personal diet, living habits, and health status. The controls provided their health information orally. Subsequently, the samples of peripheral blood were collected from all the participants with anticoagulant tubes containing ethylene diamine tetraacetic acid (EDTA).

This case–control study fully complied with the Declaration of Helsinki and local laws, and was approved by the Biomedical Ethics Committee of Anhui Medical University (demonstration report number: 20150117). After signing the written informed consent, researchers were forbidden from accessing any identity information of the participants.

### DNA preparation and genotyping

The extraction of genomic DNA from the specimens of blood was carried out with a QIAamp DNA Blood Mini Kit (Qiagen, Germany) according to the manufacturer’s protocols. The prepared DNA solutions were cryopreserved in a -80ºC refrigerator until testing. The selection of SNPs for genotyping was accomplished based on their frequencies in the Oriental population and their roles in the determination of HBGAs (Table 1). Genotyping was conducted using a patented method named SNPscan [22], which was based on double-ligation reactions and a multiplex fluorescence polymerase chain reaction (PCR), with a custom-made 48-Plex SNPscan Kit (Genesky, China). The PCR products were subsequently separated and detected with an ABI 3730 XL sequencer (ABI, Carlsbad, CA 92008, USA) using capillary electrophoresis. To meet the technical requirements of the SNPscan test, the complementary bases of rs28362459 and rs812936 were actually tested.

**Table 1:** SNP information and primer sequences used for genotyping.

SNP	Gene	Chromosome	Position	Primer	
rs1047781 (385A>T)	*FUT2*	19	48703374	AR-	CGGACGTACTCCCCCGGCAT
				TR-	CGGACGTACTCCCCCGGCAA
				3R-	GTGGCGGTATTCCTCCTCCA
rs1800028 (571C>T)	*FUT2*	19	48703560	CR-	GCATGACATGGACATAGTCCCCACG
				TR-	GCATGACATGGACATAGTCCCCACA
				3R-	GCGAACATGGACCCCTACAA
rs1800030 (849G>A)	*FUT2*	19	48703838	GF-	TTGGGACGTTCGGGATCAGG
				AF-	TTGGGACGTTCGGGATCAGA
				3F-	GCCGCATACCTCACGGGCGG
rs28362459 (59T>G)	*FUT3*	19	5844781	AR-	CGCCGCTGTCTGGCCGCTCT
				CR-	CGCCGCTGTCTGGCCGCTCG
				3R-	GCTATTTCAGCTGCTGGTGGC
rs812936 (202T>C)	*FUT3*	19	5844638	AF-	GGATGTGGAAAGGCCATGTGCA
				GF-	GGATGTGGAAAGGCCATGTACG
				3F-	TAGCAGGATCAGGAGGGTGGG

SNP, single-nucleotide polymorphism; *FUT 2*, fucosyltransferase 2; *FUT 3*, fucosyltransferase 3.

DNA preparation and genotyping were completed at Department of Blood Transfusion, the First Affiliated Hospital of USTC. For quality control, 4% of randomly chosen DNA samples were genotyped repeatedly at Genesky Biotechnologies Inc., Shanghai, China.

According to the principle of blind test, all the samples were randomly coded with a test number before DNA extraction. No participant’s information for any specimens was available until the data analysis.

### Inference of phenotype

According to the Blood Group Mutation Database (archived now) [23] and the blood typing data of local patients, a sample with any variant of rs1047781-TT, rs1800028-TT, or rs1800030-AA was classified as non-secretor (*se*), otherwise as secretor (*Se*). Similarly, an individual with rs28362459-GG or rs812936-CC was sorted as Lewis negative (*le*), otherwise as Lewis positive (*Le*). Lewis serotypes were inferred as follows: Lewis-negative individuals, both secretors and non-secretors, were classified as Le(a-b-). Lewis-positive secretors were sorted as Le(a-b+), and Lewis-positive non-secretors as Le(a+b-).

### Data analyses

The questionnaire and the genotyping data of all the participants were collected, input into a database, and proofread twice. The frequencies of each genotype, allele, secretor status, Lewis status, serotype, and haplotype were compared between the cases and the controls. Potential interactions among these factors, age, and sex were also analyzed. The effects of *FUT2/FUT3* polymorphisms on the Bath ankylosing spondylitis disease activity index (BASDAI) and the Bath ankylosing spondylitis functional index (BASFI), which were usually used to measure the disease severity of AS, were assessed specially.

### Statistical analyses

Quantitative variables with normal distribution were reported as mean ± standard deviation (SD); otherwise, as median (interquartile range, IQR). The frequency differences in sex, genotype, allele, and phenotype between the cases and the controls were all analyzed using the chi-square test. The difference in age was analyzed using the independent samples *t* test. The associations between *FUT2/FUT3* and clinical indexes of AS were analyzed using the Mann–Whitney *U* test for two independent samples and the Kruskal–Wallis test for *K* independent samples. If there was no statistical difference in a variable between the treated and untreated patients, the two parts of data would be combined. The cases with missing values were excluded test-by-test according to the results of the Missing Value Analysis. Two-factor interactions on a multiplicative scale were assessed employing the binary logistic regression. All these analyses were completed using SPSS Statistics 23.0 (IBM, USA).

The analyses of two-factor interaction on an additive scale were carried out adopting the indexes proposed by Rothman and Hosmer [24, 25]. The estimations of relative excess risk due to interaction (RERI), attributable proportion due to interaction (AP), and synergy index (S) were accomplished using the Excel sheet written by Andersson et al [26]. If the confidence interval (CI) of RERI and AP did not contain 0, and the CI of S did not include 1, it was regarded as having additive interaction between the two factors.

Multifactor interaction analyses were performed using Multifactor Dimensionality Reduction (MDR) 3.0.2. Haplotype analyses and Hardy–Weinberg equilibrium tests were conducted using Haploview 4.2. *P* values of haplotype analyses were corrected for multiple testing bias using permutation test. Sample size estimation and power analyses were conducted using PASS 11.0.7. *P* values of other multiple comparisons were all corrected adopting the false discovery rate (FDR) method using R 3.6.2. *P* or corrected *P* (if applicable) <0.05 was regarded as statistically significant.

## Results

### Demographic and clinical characteristics of participants

A total of 925 patients with AS were enlisted and 673 of them (546 males and 127 females) agreed to join this study eventually. Meanwhile, 687 (560 males and 127 females) of 892 eligible healthy controls consented to participate (control/case ≈ 1.021). The male/female ratio (4.30 vs 4.41, ***χ***^**2**^ **=** 0.856, *P* = 0.890) and the age [26.0 (11.0) vs 27.0 (10.0) years, *Z* = -1.118, *P* = 0.264] of the cases and controls were comparable (S1 Table). Furthermore, the genotype frequencies of all the five SNPs included in this study conformed to Hardy–Weinberg equilibrium (all *P* > 0.05). Raw data of genotyping outcomes were partly provided in S1 Fig, and main lifestyles and clinical characteristics of the patients with AS were listed in S1 Table.

### Associations between *FUT2*/*FUT3* polymorphisms and human susceptibility to AS

The genotype frequencies of the five SNPs included in this study showed no differences between the cases and the controls, both in the total participants and in either sex subgroup (all *P*_corrected_ > 0.05) (Table 2). At the allele level, however, a significant frequency difference of rs28362459 was observed between the patients with AS and the healthy controls among the total participants [*χ*^2^ = 7.515, *P*_corrected_ = 0.030; adjusted odds ratio (OR_adjusted_)_G/T_ = 0.782; 95% CI, 0.650–0.941; Power = 0.8] (Table 2, S2 Fig). Rs28362459-G, which usually leads to a Lewis-negative phenotype, showed a lower frequency in the patients with AS than in the healthy controls (20.3% vs 24.7%). No differences in allele frequency were found in the remaining SNPs among the total participants (all *P*_corrected_ > 0.05). In the male and female subgroups, all the five SNPs showed no significant differences in allele frequency between the cases and the controls (all *P*_corrected_ > 0.05). These suggested that the polymorphisms of rs28362459 were probably associated with human susceptibility to AS, and the allele rs28362459-G might be a protective factor for AS.

**Table 2:** Comparisons of genotype/allele frequency between cases and controls in the total participants.

Genotype/Allele		Case [n (%)]		Control [n (%)]		*χ*^2^	*P*/*P*_corrected_ value^a^		OR_adjusted_ (95.0% CI)^b^		
rs1047781	AA	216	(32.1)	219	(31.9)	2.467	0.291	/0.728			
	AT	333	(49.5)	319	(46.4)				1.043	(0.816–	1.334)
	TT	124	(18.4)	149	(21.7)				0.877	(0.644–	1.193)
	A	765	(56.8)	757	(55.1)	0.836	0.361	/0.903			
	T	581	(43.2)	617	(44.9)				0.975	(0.835–	1.140)
rs1800028	CC	665	(98.8)	680	(99.0)	0.090	0.764	/0.955			
	CT	8	(1.2)	7	(1.0)				1.110	(0.398–	3.092)
	C	1338	(99.4)	1367	(99.5)	0.089	0.765	/0.956			
	T	8	(0.6)	7	(0.5)				1.204	(0.434–	3.335)
rs1800030	GA	7	(1.0)	7	(1.0)	0.001	0.969	/0.969			
	GG	666	(99.0)	680	(99.0)				1.028	(0.354–	2.982)
	G	1339	(99.5)	1367	(99.5)	0.001	0.969	/0.969			
	A	7	(0.5)	7	(0.5)				1.073	(0.374–	3.079)
rs28362459	TT	429	(63.7)	393	(57.2)	7.384	0.025	/0.125			
	GT	215	(31.9)	249	(36.2)				0.795	(0.632–	0.996)
	GG	29	(4.3)	45	(6.6)				0.608	(0.373–	0.990)
	T	1073	(79.7)	1035	(75.3)	7.515	**0.006**	**/0.030**			
	G	273	(20.3)	339	(24.7)				0.782	(0.650–	0.941)
rs812936	TT	625	(92.9)	642	(93.4)	1.128	0.569	/0.948			
	CT	47	(7.0)	45	(6.6)				1.045	(0.683–	1.601)
	CC	1	(0.1)	0	(0.0)					---	
	T	1297	(96.4)	1329	(96.7)	0.272	0.602	/0.956			
	C	49	(3.6)	45	(3.3)				1.162	(0.768–	1.757)

^a^ *P*_corrected_ value (*P* value corrected using the FDR method). Boldface indicates statistical significance.

^b^ OR_adjusted_ (crude OR adjusted for age, sex, and the remaining SNPs). OR, odds ratio; CI, confidence interval.

Haplotype analyses of the total participants revealed significant differences between the cases and the controls in the frequencies of haplotype TT (rs812936–rs28362459) (*χ*^2^ = 5.663, *P*_permutation_ = 0.039; Power = 0.7; D' = 0.931) and TG (rs812936–rs28362459) (*χ*^2^ = 7.456, *P*_permutation_ = 0.013; Power = 0.8; D' = 0.931). A significant difference in the frequencies of haplotype TG (rs812936-rs28362459) was also found between the two groups in the female subgroup (χ^2^ = 5.624, *P*_permutation_ = 0.047; Power = 0.7; D' = 0.916) (Table 3). These results further suggested a link between *FUT3* polymorphisms and the susceptibility to AS.

**Table 3:** Frequency differences in haplotypes between cases and controls.

Group	Block	Haplotype	Frequency	Case ratio	Control ratio	*χ*^2^	*P*/*P*_permutation_ value^a^
Total	rs1047781–rs1800028–rs1800030	ACG	0.549	750.0:596.0	743.0:631.0	0.743	0.389/0.794
		TCG	0.440	581.0:765.0	617.0:757.0	0.836	0.361/0.756
	rs812936–rs28362459	TT	0.744	1028.5:317.5	995.2:378.8	5.663	**0.017/0.039**
		TG	0.221	268.5:1077.5	333.8:1040.2	7.456	**0.006/0.013**
		CT	0.031	44.5:1301.5	39.8:1334.2	0.375	0.540/0.916
Male	rs1047781–rs1800028–rs1800030	ACG	0.549	608.0:484.0	606.0:514.0	0.551	0.458/0.853
		TCG	0.441	474.0:618.0	501.0:619.0	0.394	0.530/0.909
	rs812936–rs28362459	TT	0.741	828.1:263.9	812.0:308.0	3.198	0.074/0.227
		TG	0.223	224.9:867.1	269.0:851.0	3.727	0.054/0.163
		CT	0.033	36.9:1055.1	37.0:1083.0	0.011	0.917/1.000
Female	rs1047781–rs1800028–rs1800030	ACG	0.553	144.0:110.0	137.0:117.0	0.390	0.532/0.931
		TCG	0.435	105.0:149.0	116.0:138.0	0.969	0.325/0.715
	rs812936–rs28362459	TT	0.752	200.1:53.9	182.1:71.9	3.424	0.064/0.170
		TG	0.216	43.9:210.1	65.9:188.1	5.624	**0.018/0.047**
		CT	0.023	7.9:246.1	3.9:250.1	1.390	0.238/0.583

^a^ *P*_permutation_ value (P value of permutation test). Boldface indicates statistical significance.

**Fig 1:**
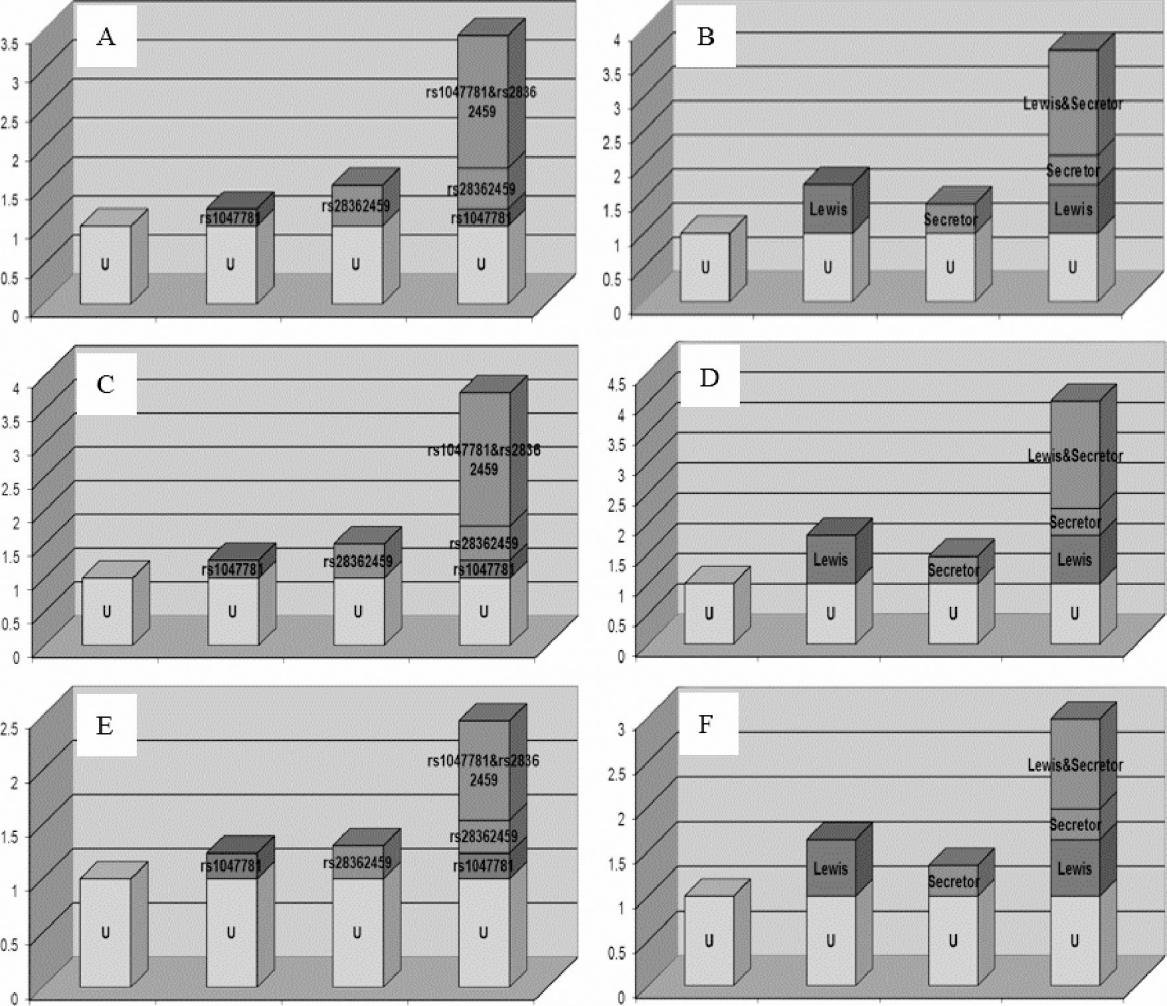
Additive interactions between rs1047781 and rs28362459, and Lewis status and secretor status. (A, C, and E) Interactions on an additive scale between rs1047781and rs28362459 for total participants, male subgroup, and female subgroup, respectively. (B, D, and F) Interactions on an additive scale between Lewis status and secretor status for the total participants, the male subgroup, and the female subgroup, respectively.

In the analyses of two-factor interaction, multiplicative interactions were found in the total participants between rs28362459-TG and age (OR_adjusted_ = 0.071; 95% CI, 0.005–0.980; *P*_corrected_ = 0.048), and rs28362459-TG and sex (OR_adjusted_ = 0.549; 95% CI, 0.305–0.989; *P*_corrected_ = 0.046). In the male subgroup, a multiplicative interaction was observed between rs28362459-TG and age (OR_adjusted_ = 0.032; 95% CI, 0.002–0.616; *P*_corrected_ = 0.022). In the female subgroup, no multiplicative interactions were found (all *P*_corrected_ > 0.05). Moreover, additive interactions were observed between rs1047781 and rs28362459 in the total participants (RERI, 0.235–3.167; AP, 0.226–0.762; S, 1.109–9.792), the male subgroup (RERI, 0.669–3.282; AP, 0.327–0.727; S, 1.246–10.173), and the female subgroup (RERI, 0.334–1.499; AP, 0.181–0.565; S, 1.001–7.251) (Fig 1A, 1C and 1E). No multifactor interactions were found in the total participants and either subgroup (all *P* > 0.05).

### Association between *FUT2*/*FUT3* phenotypes and human susceptibility to AS

At the phenotypic level, no frequency differences in the secretor status were found between the cases and the controls, whether in the total participants or either sex subgroup (all *P*_corrected_ > 0.05). Similarly, no differences were observed between the patients and the controls in terms of the frequencies of the Lewis status (all *P*_corrected_ > 0.05) and the Lewis serotype (all *P*_corrected_ > 0.05) (Table 4).

**Table 4:** Frequency differences in *FUT2/FUT3* phenotypes between cases and controls.

Participant	Phenotype/Serotype	Case [n (%)]		Control [n (%)]		*χ*^2^	*P*/*P*_corrected_ value^a^		ORadjusted (95.0% CI)^b^		
Total	*se*	124	(18.4)	149	(21.7)	2.257	0.133	/0.160			
	*Se*	549	(81.6)	538	(78.3)				1.201	(0.919–	1.569)
	*le*	30	(4.5)	45	(6.6)	2.857	0.091	/0.160			
	*Le*	643	(95.5)	642	(93.4)				1.474	(0.916–	2.372)
	Le(a-b+)	524	(77.9)	503	(73.2)	4.836	0.089	/0.160			
	Le(a+b-)	119	(17.7)	139	(20.2)				0.841	(0.639–	1.107)
	Le(a-b-)	30	(4.5)	45	(6.6)				0.656	(0.406–	1.058)
Male	*se*	98	(17.9)	123	(22.0)	2.788	0.095	/0.180			
	*Se*	448	(82.1)	437	(78.0)				1.278	(0.948–	1.721)
	*le*	27	(4.9)	39	(7.0)	2.009	0.156	/0.180			
	*Le*	519	(95.1)	521	(93.0)				1.427	(0.860–	2.368)
	Le(a-b+)	425	(77.8)	406	(72.5)	4.550	0.103	/0.180			
	Le(a+b-)	94	(17.2)	115	(20.5)				0.983	(0.684–	1.412)
	Le(a-b-)	27	(4.9)	39	(7.0)				0.830	(0.448–	1.540)
Female	*se*	26	(20.5)	26	(20.5)	0.000	1.000	/1.000			
	*Se*	101	(79.5)	101	(79.5)				0.960	(0.517–	1.784)
	*le*	3	(2.4)	6	(4.7)	1.037	0.309	/1.000			
	*Le*	124	(97.6)	121	(95.3)				1.657	(0.400–	6.869)
	Le(a-b+)	99	(78.0)	97	(76.4)	1.041	0.594	/1.000			
	Le(a+b-)	25	(19.7)	24	(18.9)				1.043	(0.554–	1.966)
	Le(a-b-)	3	(2.4)	6	(4.7)				0.612	(0.147–	2.543)

^a^ *P*_corrected_ value (*P* value corrected using the FDR method).

^b^ OR_adjusted_ (crude OR adjusted for age, sex, and the remaining factors). OR, odds ratio; CI, confidence interval.

No multiplicative interactions were found at phenotypic level in all the factors (all *P*_corrected_ > 0.05). Additive interactions were found between the Lewis status and the secretor status in the total participants (RERI, 0.747–2.338; AP, 0.247–0.590; S, 1.225–4.513), the male subgroup (RERI, 0.840–2.706; AP, 0.272–0.610; S, 1.285–4.567), and the female subgroup (RERI, 0.396–1.614; AP, 0.147–0.528; S, 1.031–4.009) (Fig 1B, 1D and 1F). No multifactor interactions were found at phenotypic level (all *P* > 0.05). These findings suggested that the phenotypes of *FUT2* and *FUT3*, and their co-determined Lewis serotypes, might not correlate with the predisposition to AS.

### Correlations between *FUT2*/*FUT3* polymorphisms and BASFI/BASDAI

To explore the links between the gene polymorphisms and the clinical manifestations of AS, association analyses were conducted at the gene and phenotypic levels. No associations were found between the gene polymorphisms and the BASFI/BASDAI (all *P*_corrected_ > 0.05). However, at the item level, the results revealed that rs1800030-A was related to an alleviated morning stiffness (the sixth item of the BASDAI, BASDAI 6; BASDAI 6_GA_: BASDAI 6_GG_ = 0.0: 1.0; *Z* = -2.575, *P*_corrected_ = 0.045; Power = 1.0), while rs28362459-G was related to a severer limitation with regard to standing without support (the sixth question of the BASFI, BASFI 6) among patients with AS (BASFI 6_GG_: BASFI 6_GT_: BASFI 6_TT_ = 1.0: 0.0: 0.0; *χ*^2^ = 10.089, *P*_corrected_ = 0.030; Power = 0.8). Lewis-positive (*Le*) patients suffered less functional loss compared with Lewis-negative (*le*) patients in terms of BASFI 6 (*Z* = -2.319, *P*_corrected_ = 0.040; Power = 0.3) and bending forward from the waist (BASFI 2; Z = -2.333, *P*_corrected_ = 0.040; Power = 0.6) (Table 5). In line with this, both Le(a+b-) and Le(a-b+) groups had less functional limitations in BASFI 6 (*χ*^2^ = 7.220, *P*_corrected_ = 0.027; Power = 0.3) and BASFI 2 (*χ*^2^ = 6.419, *P*_corrected_ = 0.040; Power = 0.5) compared with the Le(a-b-) patients. In addition, the Le(a+b-) and Le(a-b+) patients also suffered less than the Le(a-b-) ones in regard to reaching up to a high shelf (BASFI 3; *χ*^2^ = 6.848, *P*_corrected_ = 0.033; Power = 0.5) and climbing 12–15 steps independently (BASFI 7; *χ*^2^ = 6.800, *P*_corrected_ = 0.033; Power = 0.3) (Table 5). No significant differences were observed between the remaining factors and clinical variables (all *P*_corrected_ > 0.05). These results indicated that the polymorphisms of *FUT2* and *FUT3* were likely to be associated with the symptoms of AS, and rs28362459-G might aggravate relevant symptoms.

**Table 5:** Associations between *FUT2/FUT3* gene polymorphisms and the indexes of AS.

Factor	Variable(description)^a^	Subgroup	*n*	Variable value [median (IQR)]	*Z* /*χ*^2^^b^	*P*/*P*_corrected_ value^c^	
rs1800030	BASDAI 6 (Morning stiffness severity)	GG	665	1.0 (4.0)	–2.575	**0.009**	**/0.045**
		GA	7	0.0(0.0)			
rs28362459	BASFI 6 (Standing unsupported for 10 min without discomfort)	TT	429	0.0(2.0)	10.089	**0.006**	**/0.030**
		GT	215	0.0(3.0)			
		GG	29	1.0(4.0)			
Lewis	BASFI 2 (Bending forward from the waist to pick up a pen from the floor without an aid)	*le*	30	2.0(6.2)	–2.333	**0.020**	**/0.040**
		*Le*	643	0.0(4.0)			
	BASFI 6 (Standing unsupported for 10 min without discomfort)	*le*	30	1.0(4.0)	–2.319	**0.020**	**/0.040**
		*Le*	643	0.0(2.0)			
Serotype	BASFI 2 (Bending forward from the waist to pick up a pen from the floor without an aid)	Le(a-b+)	524	0.0(4.0)	6.419	**0.040**	**/0.040**
		Le(a+b-)	119	0.0(4.0)			
		Le(a-b-)	30	2.0(6.2)			
	BASFI 3 (Reaching up to a high shelf without help or aids)	Le(a-b+)	514	0.0(2.0)	6.848	**0.033**	**/0.033**
		Le(a+b-)	118	0.0(2.0)			
		Le(a-b-)	30	1.5(4.2)			
	BASFI 6 (Standing unsupported for 10 min without discomfort)	Le(a-b+)	524	0.0(2.0)	7.220	**0.027**	**/0.027**
		Le(a+b-)	119	0.0(2.0)			
		Le(a-b-)	30	1.0(4.0)			
	BASFI 7 (climbing 12–15 steps without using a handrail or walking aid)	Le(a-b+)	524	0.0(2.0)	6.800	**0.033**	**/0.033**
		Le(a+b-)	119	0.0(2.0)			
		Le(a-b-)	30	1.0(4.0)			

^a^ The clinical manifestations of ankylosing spondylitis were presented as one of the items of Bath ankylosing spondylitis disease activity index (BASDAI) or Bath ankylosing spondylitis functional index (BASFI) and the corresponding description.

^b^ *Z* and *χ*^2^ were statistical outcomes of the Mann–Whitney *U* test and the Kruskal–Wallis test, respectively.

^c^ *P*_corrected_ value (*P* value corrected using the FDR method). Boldface indicates statistical significance. AS, ankylosing spondylitis; IQR, interquartile range.

## Discussion

In view of a large degree of overlap between the susceptibility genes of IBD and AS [1, 7, 12, 13], and a strong correlation between *FUT2*/*FUT3* and IBD [16, 20, 21], it was hypothesized that a link existed between the two genes and human susceptibility to AS. The results of this study suggested that *FUT3* polymorphisms were associated with the susceptibility to AS, and rs28362459-G might be a protective factor for AS. These findings suggested that some pathogenic factors might attach to Lewis antigens in the development of AS, and the lack of these antigens might block the process. This was consistent with the report that the adhesion of *Helicobacter pylori* to gastric mucosa correlated closely with Le^b^ in the host body, and the binding of *Escherichia coli* to the epithelial cells of intestinal mucosa needed a glycosylation structure [16, 18]. This might also be similar to the situation in which infection rates of norovirus, rotavirus, and *Helicobacter pylori* were commonly much lower in non-secretors than in secretors [20, 27, 28].

The results of the haplotype analyses revealed significant differences between the cases and the controls in the frequencies of haplotypes TT (rs812936–rs28362459) and TG (rs812936–rs28362459). Both the SNPs located in *FUT3* gene, indicating associations between the gene and AS at the haplotype level. These were consistent with the results at the allele level. Although no reports on the roles of these haplotypes can be found so far, the following points may provide a rational explanation for the results: It is clear the haplotype TG (rs812936–rs28362459) is one of the determinants of Lewis-negative status; and it is supposed that the haplotype TT (rs812936–rs28362459) may influence the structure and quantity of Lewis antigens.

This study discovered a potential susceptibility gene for AS, providing fresh data for exploring the etiology of AS. Meanwhile, this was the first report of a link between a blood group gene and AS, leading to new developments in the functional study of human blood groups. Although Shinebaum reported associations between ABO antigens of non-secretors and host susceptibility to spondyloarthropathies [29], the results proved to be false later [30]. Another study also suggested that the secretor status did not correlate with AS [31].

No significant differences were found between the cases and the controls in the frequencies of secretor status, Lewis status, and Lewis serotype. However, interactions on an additive scale were found between the Lewis status and the secretor status. The interactions might reflect the fact that the two factors jointly determined the type of Lewis HBGAs, which were likely the real molecules participating in the pathogenesis of AS. Moreover, the interaction between the *FUT3* polymorphisms and age was possibly related to the influence of age on the expression of Lewis antigens, and the influence might also be associated with the maturity of human gut microbiota [18, 19].

The possible reasons for not finding correlations between *FUT3* and AS at the phenotypic level were as follows. First, both *FUT2* and *FUT3* directly encode fucosyltransferase, which may have other biological functions besides determining the human secretor status and Lewis antigens. These functions may play varied roles in the development of AS. Second, the synthesis of the Lewis HBGAs complex, such as ALe^b^, is also influenced by *FUT1* and *ABO* genes, besides being controlled by *FUT3*. Finally, the Lewis serotypes in this study were inferred only from genotyping results, with no verifications using serological and salivary tests due to specimen limitations.

This study found multiple associations between rs28362459 and the clinical manifestations of AS at the genetic and phenotypic levels. Generally speaking, rs28362459-G, Lewis-negative status, and Le(a-b-) serotype might correlate with several aggravated disease indexes. These demonstrated to some extent that disease activity, functional impairments, and imaging changes in AS were also highly heritable [1]. As mentioned earlier, rs28362459-G might undermine the development of AS, but it was also associated with some severer clinical manifestations. These seemingly inconsistent results indicated that *FUT3* might play complicated roles in the pathogenesis of AS. A probable cause for the results was that rs28362459-G might bring about multiple changes in human gut microbiota, and further affect host immune functions. This could be similar to the situation in which the intestinal flora in a non-secretor’s colon changed significantly in both structure and function among patients with CD [32, 33].

As with other case–control studies, this study also had some shortcomings. Correlations were found only between the genes and AS, but relevant mechanisms were not explored. This study involved only *FUT2* and *FUT3* genes, but did not include *FUT1* and *ABO* genes, which were also related to HBGAs. Therefore, the results of this study had certain one-sidedness. Moreover, due to the limited sample size, the power of analysis was affected to some extent for some subgroups. Due to the limited quality of questionnaire data, some analyses could not be carried out, such as the correlations between *FUT3* and relevant laboratory variables. Due to the limited size of untreated cases, the combination of treated and untreated cases, without statistical difference in nonparametric tests, was used in the analyses of associations between the gene polymorphisms and disease indexes. This might bring some bias.

To verify the findings of this study and further uncover the underlying mechanisms, researchers can investigate the compositional and functional characteristics of intestinal flora among patients with AS in the future. Furthermore, they can establish proper animal models of AS and explore the roles of HBGAs by blocking the expression of *FUT2* or *FUT3*. The findings may help in deepening the understanding of the associations between the *FUT3* gene and AS, and also in developing new treatment strategy for AS.

## Conclusions

*FUT3* gene polymorphisms correlated with human susceptibility to AS, both at the allele and haplotype level. Rs28362459-G might decrease the susceptibility, but enhance part of disease indexes of AS. This study highlighted the probability of wide associations between the blood group antigens and human autoimmune diseases.

## Supporting information

S1 Checklist STROBE statement—checklist of items that should be included in reports of observational studies. (DOCX)

S1 Fig Genotyping results of all the analyzed SNPs. (PDF)

S2 Fig Genotype frequencies (left) and allele frequencies (right) of rs28362459 between patients with AS and healthy controls. (PDF)

S1 Table Demographic and clinical characteristics of total participants. (PDF)

S1 File Questionnaire for AS in Chinese. (PDF)

S2 File Questionnaire for AS in English. (PDF)

S1 Database (XLSX)

## References

[1] Brown MA, Kenna T, Wordsworth BP. Genetics of ankylosing spondylitis—insights into pathogenesis. Nat Rev Rheumatol. 2016; 12(2):81–91. 10.1038/nrrheum.2015.133 26439405

[2] Tsui FW, Tsui HW, Akram A, Haroon N, Inman RD. The genetic basis of ankylosing spondylitis: new insights into disease pathogenesis. Appl Clin Genet. 2014; 7:105–115. 10.2147/TACG.S37325 24971029 PMC4070859

[3] Mathieu A, Paladini F, Vacca A, Cauli A, Fiorillo MT, Sorrentino R. The interplay between the geographic distribution of HLA-B27 alleles and their role in infectious and autoimmune diseases: a unifying hypothesis. Autoimmun Rev. 2009; 8(5):420–425. 10.1016/j.autrev.2009.01.003 19185064

[4] Mathieu A, Cauli A, Fiorillo MT, Sorrentino R. HLA-B27 and ankylosing spondylitis geographic distribution as the result of a genetic selection induced by malaria endemic? A review supporting the hypothesis. Autoimmun Rev. 2008; 7(5):398–403. 10.1016/j.autrev.2008.03.013 18486928

[5] Lopez-Larrea C, Sujirachato K, Mehra NK, Chiewsilp P, Isarangkura D, Kanga U, et al HLA-B27 subtypes in Asian patients with ankylosing spondylitis. Evidence for new associations. Tissue Antigens. 1995; 45(3):169–176. 10.1111/j.1399-0039.1995.tb02436.x 7761976

[6] D'Amato M, Fiorillo MT, Galeazzi M, Martinetti M, Amoroso A, Sorrentino R. Frequency of the new HLA-B*2709 allele in ankylosing spondylitis patients and healthy individuals. Dis Markers. 1995; 12(3):215–217. 10.1155/1994/394509 8590548

[7] International Genetics of Ankylosing Spondylitis C, Cortes A, Hadler J, Pointon JP, Robinson PC, Karaderi T, et al Identification of multiple risk variants for ankylosing spondylitis through high-density genotyping of immune-related loci. Nat Genet. 2013; 45(7):730–738. 10.1038/ng.2667 23749187 PMC3757343

[8] Van Praet L, Van den Bosch FE, Jacques P, Carron P, Jans L, Colman R, et al Microscopic gut inflammation in axial spondyloarthritis: a multiparametric predictive model. Ann Rheum Dis. 2013; 72(3):414–417. 10.1136/annrheumdis-2012-202135 23139267

[9] Costello ME, Ciccia F, Willner D, Warrington N, Robinson PC, Gardiner B, et al Brief Report: Intestinal Dysbiosis in Ankylosing Spondylitis. Arthritis Rheumatol. 2015; 67(3):686–691. 10.1002/art.38967 25417597

[10] Bremander A, Petersson IF, Bergman S, Englund M. Population based estimates of common comorbidities and cardiovascular disease in ankylosing spondylitis. Arthritis Care Res (Hoboken). 2011; 63(4):550–556. 21452267 10.1002/acr.20408

[11] Thjodleifsson B, Geirsson AJ, Bjornsson S, Bjarnason I. A common genetic background for inflammatory bowel disease and ankylosing spondylitis: a genealogic study in Iceland. Arthritis Rheum. 2007; 56(8):2633–2639. 10.1002/art.22812 17665420

[12] Jostins L, Ripke S, Weersma RK, Duerr RH, McGovern DP, Hui KY, et al Host-microbe interactions have shaped the genetic architecture of inflammatory bowel disease. Nature. 2012; 491(7422):119–124. 10.1038/nature11582 23128233 PMC3491803

[13] Parkes M, Cortes A, van Heel DA, Brown MA. Genetic insights into common pathways and complex relationships among immune-mediated diseases. Nat Rev Genet. 2013; 14(9):661–673. 10.1038/nrg3502 23917628

[14] O'Rielly DD, Uddin M, Rahman P. Ankylosing spondylitis: beyond genome-wide association studies. Curr Opin Rheumatol. 2016; 28(4):337–345. 10.1097/BOR.0000000000000297 27224740

[15] Grubb R. Observations on the human group system Lewis. Acta Pathol Microbiol Scand. 1951; 28(1):61–81. 10.1111/j.1699-0463.1951.tb05004.x 14818817

[16] Maroni L, van de Graaf SF, Hohenester SD, Oude Elferink RP, Beuers U. Fucosyltransferase 2: a genetic risk factor for primary sclerosing cholangitis and Crohn's disease—a comprehensive review. Clin Rev Allergy Immunol. 2015; 48(2–3):182–191. 10.1007/s12016-014-8423-1 24828903

[17] Nanthakumar NN, Dai D, Newburg DS, Walker WA. The role of indigenous microflora in the development of murine intestinal fucosyl- and sialyltransferases. FASEB J. 2003; 17(1):44–46. 10.1096/fj.02-0031fje 12475916

[18] Cooling L. Blood Groups in Infection and Host Susceptibility. Clin Microbiol Rev. 2015; 28(3):801–870. 10.1128/CMR.00109-14 26085552 PMC4475644

[19] Mackie RI, Sghir A, Gaskins HR. Developmental microbial ecology of the neonatal gastrointestinal tract. Am J Clin Nutr. 1999; 69(5):1035S–1045S. 10.1093/ajcn/69.5.1035s 10232646

[20] McGovern DP, Jones MR, Taylor KD, Marciante K, Yan X, Dubinsky M, et al Fucosyltransferase 2 (FUT2) non-secretor status is associated with Crohn's disease. Hum Mol Genet. 2010; 19(17):3468–3476. 10.1093/hmg/ddq248 20570966 PMC2916706

[21] Hu D, Zhang D, Zheng S, Guo M, Lin X, Jiang Y. Association of Ulcerative Colitis with FUT2 and FUT3 Polymorphisms in Patients from Southeast China. PLoS One. 2016; 11(1):e0146557 10.1371/journal.pone.0146557 26766790 PMC4713070

[22] Jin G, Zhu M, Yin R, Shen W, Liu J, Sun J, et al Low-frequency coding variants at 6p21.33 and 20q11.21 are associated with lung cancer risk in Chinese populations. Am J Hum Genet. 2015; 96(5):832–840. 10.1016/j.ajhg.2015.03.009 25937444 PMC4570553

[23] National Center for Biotechnology Information (NCBI). Blood Group Mutation Database [Internet]. Archived in 2017. Available from: http://ftp.ncbi.nlm.nih.gov/pub/mhc/rbc/.

[24] Rothman KJ. Epidemiology: an introduction New York: Oxford University Press; 2002.

[25] Hosmer DW, Lemeshow S. Confidence interval estimation of interaction. Epidemiology. 1992; 3(5):452–456. 10.1097/00001648-199209000-00012 1391139

[26] Andersson T, Alfredsson L, Källberg H, Zdravkovic S, Ahlbom A. Calculating measures of biological interaction. Eur J Epidemiol. 2005; 20(7):575–579. 10.1007/s10654-005-7835-x 16119429

[27] Carlsson B, Kindberg E, Buesa J, Rydell GE, Lidon MF, Montava R, et al The G428A nonsense mutation in FUT2 provides strong but not absolute protection against symptomatic GII.4 Norovirus infection. PLoS One. 2009; 4(5):e5593 10.1371/journal.pone.0005593 19440360 PMC2680586

[28] Gunaydin G, Nordgren J, Sharma S, Hammarstrom L. Association of elevated rotavirus-specific antibody titers with HBGA secretor status in Swedish individuals: The FUT2 gene as a putative susceptibility determinant for infection. Virus Res. 2016; 211:64–68. 10.1016/j.virusres.2015.10.005 26454189

[29] Shinebaum R, Blackwell CC, Forster PJ, Hurst NP, Weir DM, Nuki G. Non-secretion of ABO blood group antigens as a host susceptibility factor in the spondyloarthropathies. Br Med J (Clin Res Ed). 1987; 294(6566):208–210. 10.1136/bmj.294.6566.208 PMC1245226 3101813

[30] Smith GW, James V, Mackenzie DA, Stewart J, Blackwell CC, Elton RA, et al Ankylosing spondylitis and secretor status: a re-evaluation. Br J Rheumatol. 1997; 36(7):778–780. 10.1093/rheumatology/36.7.778 9255113

[31] Pal A, Hill M, Wordsworth P, Brown M. Secretor status and ankylosing spondylitis. J Rheumatol. 1998; 25(2):318–319. 9489826

[32] Rausch P, Rehman A, Kunzel S, Hasler R, Ott SJ, Schreiber S, et al Colonic mucosa-associated microbiota is influenced by an interaction of Crohn disease and FUT2 (Secretor) genotype. Proc Natl Acad Sci USA. 2011; 108(47):19030–19035. 10.1073/pnas.1106408108 22068912 PMC3223430

[33] Tong M, McHardy I, Ruegger P, Goudarzi M, Kashyap PC, Haritunians T, et al Reprograming of gut microbiome energy metabolism by the FUT2 Crohn's disease risk polymorphism. ISME J. 2014; 8(11):2193–2206. 10.1038/ismej.2014.64 24781901 PMC4992076

