# Supplementary material for: Associations between fucosyltransferase 3 gene polymorphisms and ankylosing spondylitis: A case–control study of an east Chinese population
Source: PLoS One. 2020 Aug 7;15(8):e0237219. doi: 10.1371/journal.pone.0237219 (PMC7413420; doi:10.1371/journal.pone.0237219)
Supplement: S1 Checklist — (DOCX) [file pone.0237219.s001.docx]

STROBE Statement—checklist of items that should be included in reports of observational studies

|  | Item No. | Recommendation | Page  No. | Relevant text from manuscript |
| --- | --- | --- | --- | --- |
| **Title and abstract** | 1 | (*a*) Indicate the study’s design with a commonly used term in the title or the abstract | 1 | case–control study |
|  |  | (*b*) Provide in the abstract an informative and balanced summary of what was done and what was found | 3 | Questionnaires of 673 patients with AS...  For rs28362459, a significant difference… |
| Introduction | | | |  |
| Background/rationale | 2 | Explain the scientific background and rationale for the investigation being reported | 4-5 | Therefore, it is promising to further search for susceptibility genes of AS among infection-related genes...  *FUT2* and *FUT3* genes are closely related to gut inflammation… |
| Objectives | 3 | State specific objectives, including any prespecified hypotheses | 5-6 | it is supposed that *FUT2*, *FUT3*, and HBGAs may play certain roles in the pathogenesis of AS…  The aim of this study was to explore the associations between… |
| Methods | | | |  |
| Study design | 4 | Present key elements of study design early in the paper | 6 | Patients with AS were selected…  This case–control study fully… |
| Setting | 5 | Describe the setting, locations, and relevant dates, including periods of recruitment, exposure, follow-up, and data collection | 6 | …the First Affiliated Hospital of Anhui Medical University from January 2015 to June 2018 |
| Participants | 6 | (*a*) *Cohort study*—Give the eligibility criteria, and the sources and methods of selection of participants. Describe methods of follow-up  *Case-control study*—Give the eligibility criteria, and the sources and methods of case ascertainment and control selection. Give the rationale for the choice of cases and controls  *Cross-sectional study*—Give the eligibility criteria, and the sources and methods of selection of participants | 6 | All the patients were diagnosed by senior rheumatologists according to the New York Diagnostic Criteria…  age- and sex-matched healthy controls…had no history of…  (to remove the confound effect of age, sex, relevant diseases ) |

Continued on next page

|  |  | (*b*) *Cohort study*—For matched studies, give matching criteria and number of exposed and unexposed  *Case-control study*—For matched studies, give matching criteria and the number of controls per case | 6  10 | …673 patients with AS…687 age- and sex-matched healthy controls…  …(control/case ≈ 1.02) |
| --- | --- | --- | --- | --- |
| Variables | 7 | Clearly define all outcomes, exposures, predictors, potential confounders, and effect modifiers. Give diagnostic criteria, if applicable | 8  6 | the frequencies of the genotype, allele…  the effects of *FUT2/FUT3* polymorphisms on…BASDAI…  New York Diagnostic Criteria (revised in 1984)… |
| Data sources/ measurement | 8* | For each variable of interest, give sources of data and details of methods of assessment (measurement). Describe comparability of assessment methods if there is more than one group | 6-8 | Cases: at the clinic of rheumatology and immunology… questionnaire…. genotyping… Inference of phenotype  Control: the Health Checkup Center… genotyping… Inference of phenotype |
| Bias | 9 | Describe any efforts to address potential sources of bias | 6  9  10 | age- and sex-matched healthy controls…had no history of…  If there was no statistical difference in a variable between the treated and untreated patients, the two parts of data would be combined.  …the cases and the controls were comparable…conformed to Hardy–Weinberg equilibrium |
| Study size | 10 | Explain how the study size was arrived at | 9 | Sample size estimation was conducted using PASS 11.0.7. |
| Quantitative variables | 11 | Explain how quantitative variables were handled in the analyses. If applicable, describe which groupings were chosen and why | 9 | Quantitative variables with normal distribution were reported as mean ± standard deviation (SD) ; otherwise, as median (interquartile range, IQR). |
| Statistical methods | 12 | (*a*) Describe all statistical methods, including those used to control for confounding | 9 | *…*chi-square test …Mann–Whitney *U* test…Kruskal–Wallis test… |
|  |  | (*b*) Describe any methods used to examine subgroups and interactions | 9-10 | logistic regression…proposed by Rothman and Hosmer…Multifactor Dimensionality Reduction (MDR) |
|  |  | (*c*) Explain how missing data were addressed | 9 | The cases with missing values were excluded test-by-test according to the results of the Missing Value Analysis. |

Continued on next page

|  |  | (*d*) *Cohort study*—If applicable, explain how loss to follow-up was addressed  *Case-control study*—If applicable, explain how matching of cases and controls was addressed  *Cross-sectional study*—If applicable, describe analytical methods taking account of sampling strategy | 6  8-9 | …687 age- and sex-matched healthy controls…  The frequencies of each genotype, allele, secretor status, Lewis status, serotype, and haplotype were compared between the cases and the controls. |
| --- | --- | --- | --- | --- |
|  |  | (*e*) Describe any sensitivity analyses | 10 | *P* (or corrected *P*, if applicable) <0.05 was regarded as statistically significant |
| Results | | | | |
| Participants | 13* | (a) Report numbers of individuals at each stage of study—eg numbers potentially eligible, examined for eligibility, confirmed eligible, included in the study, completing follow-up, and analysed | 10 | Case: A total of 925 patients with AS were enlisted and 673 of them (546 males and 127 females) agreed to join this study eventually.  Control: Meanwhile, 687 (560 males and 127 females) of 892 healthy controls consented to participate. |
|  |  | (b) Give reasons for non-participation at each stage | 10 | (Disagreement, which has been indicated in manuscript.) |
|  |  | (c) Consider use of a flow diagram |  | (There was no change in the number of participants throughout the study.) |
| Descriptive data | 14* | (a) Give characteristics of study participants (eg demographic, clinical, social) and information on exposures and potential confounders | 10 | Case: The lifestyles and clinical characteristics of the patients with AS were partly listed in S1 Table.  Control: 687 (560 males and 127 females) of 892 eligible healthy controls…The male/female ratio …and the age... |
|  |  | (b) Indicate number of participants with missing data for each variable of interest | 11-19 | (The qualified data of the cases and the controls were listed in table 2-5 and table S1 in a proper form. The missing values for each variable were indicated.) |
|  |  | (c) *Cohort study*—Summarise follow-up time (eg, average and total amount) |  | N/A |
| Outcome data | 15* | *Cohort study*—Report numbers of outcome events or summary measures over time |  | N/A |
|  |  | *Case-control study—*Report numbers in each exposure category, or summary measures of exposure | 11-19 | Table 2 -5 |
|  |  | *Cross-sectional study—*Report numbers of outcome events or summary measures |  | N/A |

Continued on next page

| Main results | 16 | (*a*) Give unadjusted estimates and, if applicable, confounder-adjusted estimates and their precision (eg, 95% confidence interval). Make clear which confounders were adjusted for and why they were included | 11-16 | Table 2 and 4 |
| --- | --- | --- | --- | --- |
|  |  | (*b*) Report category boundaries when continuous variables were categorized |  | N/A (No continuous variables were categorized.) |
|  |  | (*c*) If relevant, consider translating estimates of relative risk into absolute risk for a meaningful time period |  | N/A |
| Other analyses | 17 | Report other analyses done—eg analyses of subgroups and interactions, and sensitivity analyses | 13  14-15  17 | Haplotype analyses…  analyses of two-factor interaction…  multifactor interactions  Interaction analyses |
| Discussion | | | | |
| Key results | 18 | Summarise key results with reference to study objectives | 20 | In view of a large degree of…The results of this study suggested that *FUT3* polymorphisms were associated with the susceptibility to AS… |
| Limitations | 19 | Discuss limitations of the study, taking into account sources of potential bias or imprecision. Discuss both direction and magnitude of any potential bias | 22 | This study also had some shortcomings… |
| Interpretation | 20 | Give a cautious overall interpretation of results considering objectives, limitations, multiplicity of analyses, results from similar studies, and other relevant evidence | 20-22 | These findings suggested…  No significant differences were found between the cases and…  The possible reasons for not finding correlations…  These demonstrated to some extent that disease activity… |
| Generalisability | 21 | Discuss the generalisability (external validity) of the study results | 20 | This study discovered a potential susceptibility gene…this was the first report of a link between a blood group gene and AS… |
| Other information | |  | | |
| Funding | 22 | Give the source of funding and the role of the funders for the present study and, if applicable, for the original study on which the present article is based | 1-2 | This study was funded by… |

*Give information separately for cases and controls in case-control studies and, if applicable, for exposed and unexposed groups in cohort and cross-sectional studies.

**Note:** An Explanation and Elaboration article discusses each checklist item and gives methodological background and published examples of transparent reporting. The STROBE checklist is best used in conjunction with this article (freely available on the Web sites of PLoS Medicine at http://www.plosmedicine.org/, Annals of Internal Medicine at http://www.annals.org/, and Epidemiology at http://www.epidem.com/). Information on the STROBE Initiative is available at www.strobe-statement.org.
