# Supplementary material for: Associations between fucosyltransferase 3 gene polymorphisms and ankylosing spondylitis: A case–control study of an east Chinese population
Source: PLoS One. 2020 Aug 7;15(8):e0237219. doi: 10.1371/journal.pone.0237219 (PMC7413420; doi:10.1371/journal.pone.0237219)
Supplement: S1 Fig — (PDF) [file pone.0237219.s002.pdf]

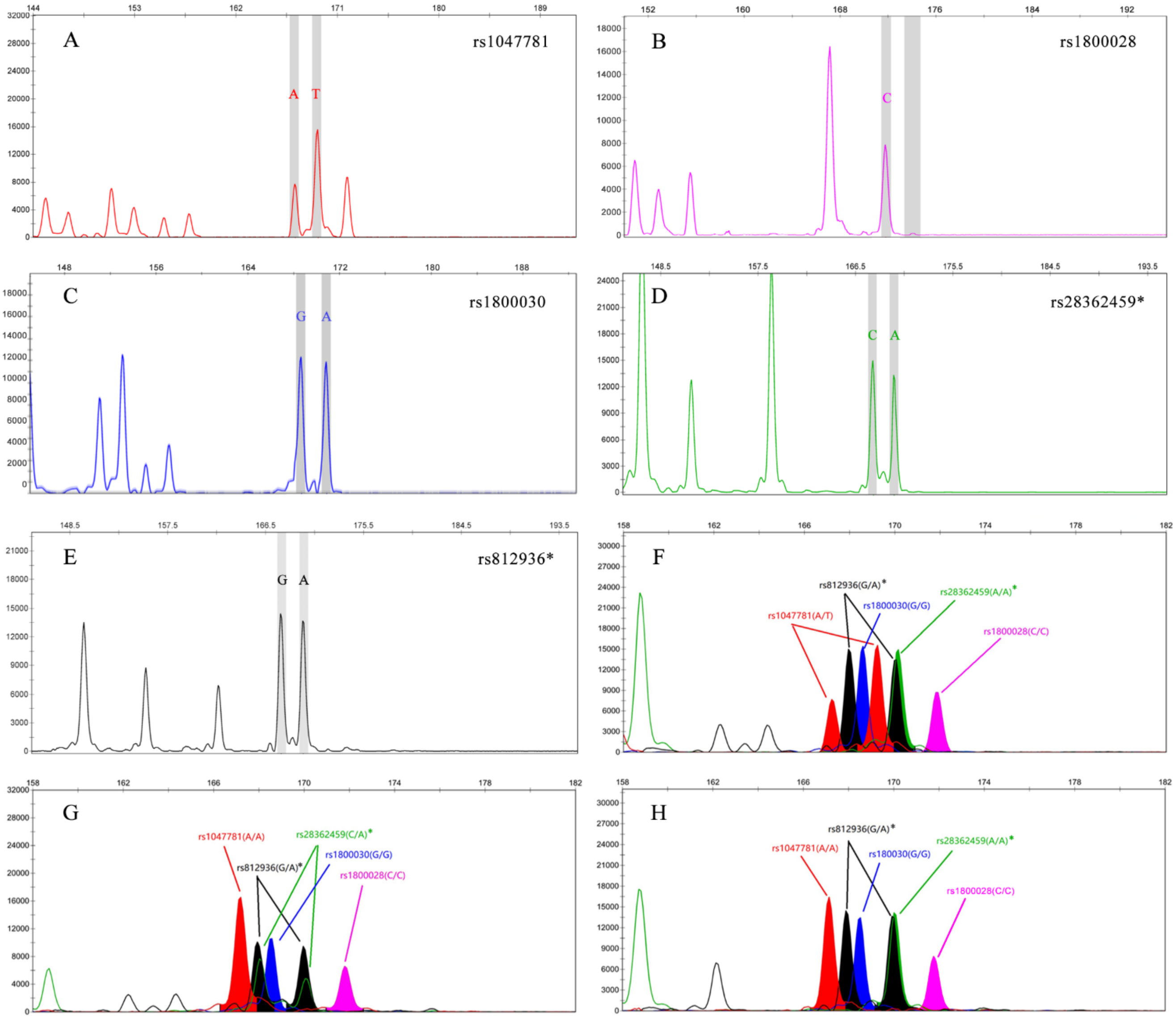

S1 Fig. Genotyping results of all the analyzed SNPs. (A-C) Raw data of PCR-sequencing outcomes for *FUT2* (rs1047781), *FUT2* (rs1800028), and *FUT2* (rs1800030) respectively. (D, E) Raw data of PCR-sequencing outcomes for *FUT3* (rs28362459) and *FUT3* (rs812936), respectively. (F-H) Superposed maps of genotyping results for single specimens (participant ID: 10930, 18850, and 452 respectively). *To meet the technical requirements of SNPscan test, the complementary bases of SNPs were actually tested.
