# Supplementary material for: Associations between fucosyltransferase 3 gene polymorphisms and ankylosing spondylitis: A case–control study of an east Chinese population
Source: PLoS One. 2020 Aug 7;15(8):e0237219. doi: 10.1371/journal.pone.0237219 (PMC7413420; doi:10.1371/journal.pone.0237219)
Supplement: S2 Fig — (PDF) [file pone.0237219.s003.pdf]

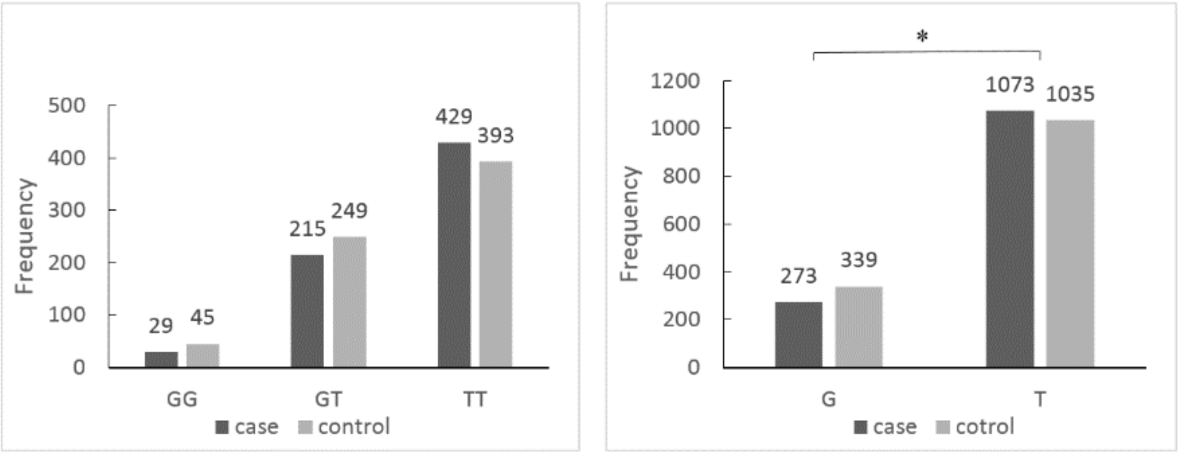

S2 Fig. Genotype frequencies (left) and allele frequencies (right) of rs28362459 between patients with AS and healthy controls. *Significant difference. OR, odds ratio; CI, confidence interval.
