# Supplementary material for: Associations between fucosyltransferase 3 gene polymorphisms and ankylosing spondylitis: A case–control study of an east Chinese population
Source: PLoS One. 2020 Aug 7;15(8):e0237219. doi: 10.1371/journal.pone.0237219 (PMC7413420; doi:10.1371/journal.pone.0237219)
Supplement: S1 Table — (PDF) [file pone.0237219.s004.pdf]

S1 Table. **Demographic and clinical characteristics of total participants.**

**Characteristics**		**Case**	**Control**	χ^2/Z^a	***P* value**
Sex (*n*, %)	Male	546（81.1）	560（81.5）	0.856	0.890
	Female	127（18.9）	127（18.5）		
Age [year, median (IQR)]		26.0(11.0)	27.0(10.0)	-1.118	0.264
Height (cm, *n* = 668, mean ± SD)		170.1 ± 6.6			
Weight (kg, *n* = 665, mean ± SD)		64.5 ± 12.7			
Smoking (*n*, %)	Nonsmoker	438 (65.1)			
	Smoker	235 (34.9)			
Drinking (*n*, %)	Nondrinker	490 (74.1)			
	Drinker	171 (25.9)			
Salt intake (*n*, %)	Low	82 (12.9)			
	Moderate	374 (58.8)			
	High	180 (28.3)			
Drinking water (*n*, %)	Underground water	146 (22.8)			
	Tap water	474 (74.2)			
	Mineral water	19 (3.0)			
Living in a cold and humid environment (*n*, %)	No	213 (80.1)			
	Yes	53 (19.9)			
Sleep quality (*n*, %)	Poor	152 (23.0)			
	Average	300 (45.5)			

	Good	208 (31.5)			
Exercise (*n*, %)	Hardly do	160 (57.1)			
	Occasionally do	83 (29.6)			
	Often do	37 (13.2)			
Exercise intensity (*n*, %)	Low	96 (64.9)			
	Moderate	50 (33.8)			
	High	2 (1.4)			
Occupation (*n*, %)	Physical labor	265 (40.1)			
	Brainwork	186 (28.1)			
	Else	210 (31.8)			
Per capita monthly income (¥, *n*, %)	<1000	15 (6.3)			
	1000~2000	50 (21.1)			
	2000~4000	110 (46.4)			
	>4000	62 (26.2)			
Family history of AS (*n*, %)	No	392 (68.9)			
	Yes	177 (31.1)			
Site of illness (*n*, %)	Axial joint	396 (62.4)			
	Peripheral joint	210 (33.1)			
	Tendonitis	3 (0.5)			
	Else	26 (4.1)			
BASFI [*n* = 662, median (IQR)]		0.9 (2.6)			
BASDAI [*n* = 672, median(IQR)]		2.0 (3.2)			

ASDAS [n = 210, median (IQR)]		3.0 (3.0)			
Schober's test [n = 673, median (IQR)]		0.0 (4.4)			
Degree of thoracic motion [n = 673, median (IQR)]		0.0 (2.5)			
Finger-floor distance [cm, n = 238, median (IQR)]		5.0 (17.0)			
Pillow-wall distance [cm, n = 673, median (IQR)]		0.0 (0.0)			
Back pain [n = 673, median (IQR)]		0.0 (0.0)			
Medical imaging grade [n = 46, median (IQR)]		3.0 (1.0)			
HLA-B27 (n, %)	Negative	254 (38.1)			
	Positive	413 (61.9)			
Having other diseases (n, %)	No	581 (86.3)			
	Yes	92 (13.7)			
Treatment within 3 months (n, %)	Never received	164 (26.4)			
	Received	458 (73.6)			

[a] χ^2 and Z values were statistical outcomes of the chi-square test for sex analysis and the Mann–Whitney U test for age analysis between the cases and the controls, respectively. SD, standard deviation; IQR, interquartile range; BASFI, Bath ankylosing spondylitis functional index; BASDAI, Bath ankylosing spondylitis disease activity index; ASDAS, ankylosing spondylitis disease activity score; HLA, human leukocyte antigen.
