# Supplementary material for: Associations between fucosyltransferase 3 gene polymorphisms and ankylosing spondylitis: A case–control study of an east Chinese population
Source: PLoS One. 2020 Aug 7;15(8):e0237219. doi: 10.1371/journal.pone.0237219 (PMC7413420; doi:10.1371/journal.pone.0237219)
Supplement: S1 File — (PDF) [file pone.0237219.s005.pdf]

ID:____________________ 编号:______________

尊敬的患者/体检者：

您好！如果您已充分知晓本研究的目的、意义及概况，并同意为了支持本研究而提供必要的健康信息和相关标本，请填写本表。填写过程中，若遇到任何问题，请向现场工作人员询问。谢谢!

强直性脊柱炎流行病学调查表（第 3 版）

1. 您是否为先证者 0. 否 1. 是

一般情况：

2. 姓名______________
3. 籍贯 ______________________
4. 常住地址____________________
5. 联系电话____________________
6. 性别： 0. 女 1. 男
7. 出生年月:______年___月___日
8. 民族： 1.汉族 2. 回族 3. 其他
9. 职业：1. 以体力劳动为主_______________
   2.以脑力劳动为主_______
   3.其他________
10. 文化程度： 1. 文盲 2. 小学 3.初中 4. 高中或中专 5. 大专及以上
11. 家庭人均月收入：
    1. <1000 元 2. 1000~2000 元
    3. 2000~4000 元 4. 4000 以上
12. 您目前每月（近六个月）因该病所要承担的经济费用：
    1. <200 元 2. 200-500 元 3. 500-1000 元
    4. 1000-5000 元 5. 5000 以上

身体状况：

13. 身高：___________cm
14. 体重：____________kg

环境因素（生活习惯）：

15. ①您既往或发病前吸烟吗？（每天吸≥1 支，持续 1 年；或总量≥18 包）
    0. 否 1. 是
    ②开始吸烟时的年龄为_____周岁
    ③既往/发病前平均每天吸_____支烟
16. ①您既往或发病前经常饮酒吗？（每周饮酒两次或两次以上）
    0. 否 1. 是

种类	是/否	饮用量	平均每周次数
A. 白酒（两）			
B. 啤酒（瓶）			
c. 其他（两）			

②开始饮酒时的年龄为________周岁
③您既往或发病前常饮什么酒?

17. 您的食盐口味： 1.淡 2.中等 3.咸
18. 烹饪用油：1. 完全植物油 2. 主要植物油 3. 植物油、动物油各半 4. 主要动物油 5. 完全动物油
19. 食肉情况： 1. 瘦肉为主 2. 肥瘦各半 3. 肥肉为主
20. 奶及奶制品食用情况： 1. 基本不食用 2. 每月 1 次 3. 每周 1 次 4. 每周 2 次 5. 每周3-4 次 6. 每天1次 7. 每天 2 次及以上
21. 您家的饮水类型是：1. 地下水 2.自来水 3.矿泉水 4.其他_________
22. 您家附近是否有高噪声源？0.否. 1.是
23. 您的睡眠质量：1. 较差 2.一般 3.较好
24. 您是否长期居住在阴冷潮湿的环境中或经常受凉？
    0. 否 1. 是
25. ①您日常体育锻炼情况？1.基本不锻炼 2. 偶尔 3. 经常
    ②运动类型： 1.体操 2.游泳 3.慢跑 4.爬山 5.球类 6.其他_________
    ③运动强度：1.低强度 2.中等强度 3.高强度

☆血清学指标:

	具体数值	“＋”或“－”	参考值范围
A. HLA-B27			
B. ESR			男：0~15mm/L；女：0~20mm/L
C. CRP			
D. WBC			$3.5\text{~}9.5\times10^9$/L
E. 中性粒细胞			$1.8\text{~}6.3\times10^9$/L
F. 抗-O			0~200IU/ml
G. RF			0~14IU/ml
H. VitD			

影像学指标：

26. 骶髂关节分级： X 片___________________
CT____________________
MRI___________________

家族史：

27. 家族中是否有 AS 患者？ 0. 否 1. 是（若有，请填写与其血缘关系）
1.__________________________________
2.__________________________________
3.__________________________________
4.__________________________________

疾病史：

28. 除 AS 外，您还在何时患过何种疾病(包括感染和手术等)？
1.__________________________________
2.__________________________________
3.__________________________________
4.__________________________________

发病情况:

29. 是否为首次发病？ 1. 首发 2. 再发
30. 首次出现症状为:__________________________________ 出现时间为： ____________
31. 首次发病部位： 1. 中轴关节 2.外周关节症状 3. 肌腱炎 4. 其他_______________
32. 是否接受过治疗？ 0. 否 1. 是
33. 首次治疗时间为:_____年___月___日; 最近治疗时间：_____年___月___日

34. 治疗情况：（“0”表示未用过或无效；“1”表示用过或有效；“9”表示不清楚）

药物类别	名称	时间及剂量	是否有效	药物类别	名称	时间及剂量	是否有效
A. 非甾体药				D. 沙利度胺	/		
B. 生物制品				F. 中成药			
C. SASP	/			G. 其他			

AS 相关指数：

☆BASFI（Bath Ankylosing Spondylitis Functional Index）

项目	VAS 得分*
BASFI 1. 无需别人帮助或辅助器材，穿袜子或贴身衣服。	
BASFI 2 无需辅助器材，向前弯腰从地上拾取钢笔。	
BASFI 3. 无需别人帮助或辅助器材，从较高的储物架上取物。	
BASFI 4. 无需用手或别人帮助，从所坐的无扶手餐桌椅上站立起来。	
BASFI 5. 无需别人帮助，从仰躺着的地板上站立起来。	
BASFI 6. 不改变姿态，无任何辅助支撑地站立 10 分钟。	
BASFI 7. 不用扶手或其他辅助器材，走 12-15 级台阶，每步一个台阶。	
BASFI 8. 不转身，从肩膀处向后看。	
BASFI 9. 完成体力活动，如理疗运动、园艺或体育运动。	
BASFI 10. 完成一整天的家务和工作。	
BASFI	

*请使用视觉模拟量表来回答测试中的问题。0 为“容易”，10 为“不可能”。BASFI 为 10 个量表得分的平均值。

☆BASDAI（Ankylosing Spondylitis Disease Activity Index）

项 目	VAS 得分*
BASDAI1 (A). 过去一周你感受到的疲劳/困倦的总体程度？	
BASDAI2 (B). 过去一周你感受到的颈痛、背痛和髋痛总体程度？	
BASDAI3 (C). 过去一周除了颈痛、背痛和髋痛外，你感受到的其他关节疼痛/肿胀的总体程度？	
BASDAI4 (D). 过去一周肌腱和韧带炎症(结缔组织嵌入骨头的局部压痛区)?	
BASDAI5 (E). 当你清醒后晨僵持续多长时间(min)？(无晨僵=0，30 分钟=2.5，60 分钟=5，90 分钟=7.5，120 分钟或以上=10)	分钟
BASDAI6 (F). 过去一周在清醒后你感受到的晨僵的总体程度?	
BASDAI=0.2*[A+B+C+D+0.5*（E/12+F）]	

*请用 0 - 10 量表来衡量不适、疼痛和疲劳程度(0 表示没有问题，10 表示问题最严重)，并回答 6 个关于 AS 五大症状的问题。

☆AS 疾病活动评分(ASDAS)

患者总体评价(PGA):______________

a. = 0.122×腰背痛+ 0.061×晨僵时间 + 0.119×患者总体评价 + 0.210×SQRT(ESR) + 0.383×ln(CRP+1);

b. = 0.079×腰背痛 + 0.069×晨僵时间 + 0.113×患者总体评价 + 0.086×外周关节疼痛/肿胀 + 0.293×SQRT(ESR);

c. = 0.121×腰背痛 + 0.058×晨僵时间 + 0.110×患者总体评价 + 0.073×外周关节疼痛/肿胀 + 0.579×ln(CRP+1) ;

d. = 0.152×腰背痛 + 0.069×晨僵时间+ 0.078×疲倦+ 0.224×SQRT(ESR)+ 0.400×ln(CRP+1).

(注：腰背痛---BASDAI2；晨僵时间---BASDAI5；外周关节疼痛/肿胀---BASDAI3；疲倦---BASDAI1)

专项检查:

疼痛程度	
列　项	结　果
总体背痛程度	cm
夜间背痛程度	cm
指地距	cm

脊柱活动程度	
列　项	结　果
胸廓活动度	cm
Schober 检查	cm
枕墙距	cm

调查员：________________________　　调查日期：________年___月___日
