# Supplementary material for: Associations between fucosyltransferase 3 gene polymorphisms and ankylosing spondylitis: A case–control study of an east Chinese population
Source: PLoS One. 2020 Aug 7;15(8):e0237219. doi: 10.1371/journal.pone.0237219 (PMC7413420; doi:10.1371/journal.pone.0237219)
Supplement: S2 File — (PDF) [file pone.0237219.s006.pdf]

ID:____________________ No.:________________

Dear patients/physical examiners:

Good morning/afternoon! If you are fully aware of the purpose, significance and profile of this study, and agree to provide necessary health information and relevant specimens to support this study, please complete this form. If you encounter any problems during the filling process, please ask the staff on site. Thank you!

Epidemiological Questionnaire for Ankylosing Spondylitis

(3rd Edition)

---

1. Are you a proband? 0. No 1. Yes

General information:

2. Name___________
3. Native place ____________
4. Address ____
5. Telephone number_______________________
6. Gender: 0. Female 1. Male
7. Date of birth ________________
8. Ethnic group: 1. Han 2. Hui 3. Other______
9. Occupation: 1. mainly physical labor 2. Mainly mental work 3. Other________
10. Education: 1. Illiteracy 2. Primary school 3. Junior high school 4. High school or technical secondary school 5. Junior college or above
11. Household per capita monthly income (RMB):
    1. <1000 yuan 2. 1000-2000 yuan 3. 2000-4000 yuan 4. >4000 yuan
12. Your monthly costs (within six months) due to AS (RMB):
    1. <200 yuan 2.200-500 yuan 3.500-1000 yuan 4. 1000-5000 yuan 5. >5000 yuan

---

Physical Information:

13. Height:________cm
14. Weight:________kg

Environmental factors (living habits):

15. Did you smoke before you developed AS? (1 cigarette or more per day for 1 year; or a total of 18 packets)
    0. No 1. Yes
    Age of starting smoking ______
    Average number of cigarettes consumed daily ____
16. Did you frequently drink alcohol before developing AS? (Drinking twice or more weekly)
    0. No 1. Yes

Type	Yes/No	Drinking volume	Average times per week
A. Liquor (liang or 50 g)			
B. Beer (bottle or 500 ml)			
C. Other (liang or 50 g)			

Age of first drinking _______

What brand of alcohol did you drink before suffering AS?____________________

17. Salt intake: 1. Light 2. Medium 3. Heavy

18. Cooking-oil intake: 1. Completely vegetable oil 2. Mainly vegetable oil 3. Vegetable and animal oil 4. Mainly animal oil 5. Completely animal oil

19. Meat intake: 1. Mainly lean meat 2. Fat and lean meat 3. Mainly fat

20. Dairy intake: 1. Hardly 2. Once a month 3. Once a week 4. Twice a week 5. 3-4 times a week 6. Once a day 7. Twice a day or more

21. Drinking water: 1. Well water 2. Tap water 3. Mineral water 4. Other ________

22. Is there a strong source of noise near your house? 0. No. 1. Yes

23. Your sleep quality: 1. Poor 2. Normal 3. Good

24. Do you usually live in a cold and humid environment or often catch cold?
0. No 1. Yes

25. How about your physical activities? 1. No exercise at all 2. Occasionally do 3. Frequently do

Type of exercise: 1. Gymnastics 2. Swimming 3. Jogging 4. Climbing 5. Balls 6. Other __________

Exercise intensity: 1. Low 2. Medium 3. High

Serological test results:

Variable	Value	+/−	Reference
A. HLA-B27			
B. ESR			Male：0~15 mm/L; Female：0~20 mm/L
C. CRP			
D. WBC			$3.5\sim9.5\times10^9$/L
E. Neutrophil			$1.8\sim6.3\times10^9$/L
F. Anti-O			0~200IU/ml
G. RF			0~14IU/ml
H. Vit.D			

Image changes:

26. Grade of sacroiliac joint changes:

X-ray ____;

CT ____;

MRI ____

Family history:

27. Are there AS patients in your family? 0. No 1. Yes (if yes, please fill in the

consanguinity)

1.________________________________

2.________________________________

3.________________________________

4.________________________________

Disease history:

28. In addition to AS, what other disease (and when) do you have? (including infection and surgery)

1.________________________________

2.________________________________

3.________________________________

4.________________________________

Status of AS:

29. Is this the first onset of AS? 0. No 1. Yes

30. The symptoms appeared at the first onset of AS : ________________________________

The date of the first onset: _______________

31. Sites affected at the first onset of AS: 1. Joint axis 2. Peripheral joints 3. Tendinitis 4. Other _______

32. Have you received any medical treatment? 0. No 1. Yes

33. The date of receiving the first treatment: _______________; The date of the latest treatment: _________________

34. Drugs used ("0" means unused or invalid; "1" means used or valid; "9" means not clear):

**Classification**	**Name of Drug**	**Using time and dose**	**Valid/invalid**	**Classification**	**Name of Drug**	**Using time and dose**	**Valid/invalid**
A. Non-steroidal drugs				D. Thalidomide	/		
B. biological product				F. Chinese Traditional medicine			
C. SASP	/			G. Other			

Relevant indexes of AS:

☆BASFI（Bath Ankylosing Spondylitis Functional Index）

**Items**	**VAS***
BASFI1. Putting on your socks or tights without help or aids?	
BASFI2. Bending forward from the waist to pick up a pen from the floor without an aid?	

BASFI3. Reaching up to a high shelf without help or aids?	
BASFI4. Getting up from an armless chair without using your hands or any other help?	
BASFI5. Getting up off the floor without any help from lying on your back?	
BASFI6. Standing unsupported for 10 minutes without discomfort?	
BASFI7. Climbing 12-15 steps without using a handrail or walking aid (one foot on each step)?	
BASFI8. Looking over your shoulder without turning your body?	
BASFI9. Doing physically demanding activities, such as physiotherapy exercises, gardening or sports?	
BASFI10. Doing a full day activities whether it be at home or work?	
BASFI	

*Please use a visual analogue scale (VAS) to answer the questions above, with 0 being "easy" and 10 being "impossible". BASFI score is the mean of the ten scales.

☆BASDAI（Ankylosing Spondylitis Disease Activity Index）

**Items**	**VAS***
BASDAI1(or A): Fatigue in the past week?	
BASDAI2 (or B): Spinal pain in the past week?	
BASDAI3 (or C): Arthralgia or joint swelling in the past week?	
BASDAI4(or D): Enthesitis or inflammation of tendons and ligaments (areas of localized tenderness where connective tissues insert into bone) in the past week?	
BASDAI5(or E): Morning stiffness duration (min)? (no morning stiffness = 0, 30 minutes = 2.5, 60 minutes = 5, 90 minutes = 7.5, ≥120 minutes = 10)	min
BASDAI6(or F): Morning stiffness severity in the past week?	
BASDAI = 0.2*[A+B+C+D+0.5*（E/12+F）]	

*Please use a 0 - 10 scale, measuring discomfort, pain, and fatigue (0 being no problem and 10 being the worst problem), to answer the six questions pertaining to the five major symptoms of AS.

☆AS Disease Activity Score (ASDAS)

Patient Global Assessment (PGA):________________

a. = 0.122×BASDAI2 + 0.061×BASDAI5 + 0.119×PGA + 0.210×SQRT(ESR) + 0.383×ln(CRP+1);

b. = 0.079×BASDAI2 + 0.069×BASDAI5 + 0.113×PGA + 0.086×BASDAI3 + 0.293×SQRT(ESR);

c. = 0.121×BASDAI2 + 0.058×BASDAI5 + 0.110×PGA + 0.073×BASDAI3 + 0.579×ln(CRP+1);

d. = 0.152×BASDAI2 + 0.069×BASDAI5+ 0.078×BASDAI1+ 0.224×SQRT(ESR) + 0.400×ln(CRP+1).

Special Examinations:

Pain severity	
**Items**	**Results**
Overall back pain	cm
Back pain at nighttime	cm
Finger-floor distance	cm

Spinal activity	
**Items**	**Results**
Thoracic motion	cm
Schober's test	cm
Pillow-wall distance	cm

Investigator:________________ Date of investigation: __________________
